# The Sorghum bicolor Root Exudate Sorgoleone Shapes Bacterial Communities and Delays Network Formation

**DOI:** 10.1128/mSystems.00749-20

**Published:** 2021-03-16

**Authors:** Peng Wang, Yen Ning Chai, Rebecca Roston, Franck E. Dayan, Daniel P. Schachtman

**Affiliations:** a University of Nebraska—Lincoln, Center for Plant Science Innovation, Department of Agronomy and Horticulture, Lincoln, Nebraska, USA; b University of Nebraska—Lincoln, Center for Plant Science Innovation, Department of Biochemistry, Lincoln, Nebraska, USA; c Colorado State University, Department of Agricultural Biology, Fort Collins, Colorado, USA; University of Dundee

**Keywords:** root exudates, nitrification, microbiome, sorghum, sorgoleone, nitrogen, soil property

## Abstract

Primary and secondary metabolites exuded from roots are key drivers of root-soil microbe interactions that contribute to the structure and function of microbial communities. Studies with model plants have begun to reveal the complex interactions between root exudates and soil microbes, but little is known about the influence of specialized exudates from crop plants. The aims of this work were to understand whether sorgoleone, a unique lipophilic secondary benzoquinone exuded only from the root hairs of sorghum, influences belowground microbial community structure in the field, to assess the effect of purified sorgoleone on the cultured bacteria from field soils, and to determine whether sorgoleone inhibits nitrification under field conditions. Studies were conducted comparing wild-type sorghum and lines with genetically reduced sorgoleone exudation. In the soil near roots and rhizosphere, sorgoleone influenced microbial community structure as measured by β-diversity and network analysis. Under greenhouse conditions, the soil nitrogen content was an important factor in determining the impacts of sorgoleone. Sorgoleone delayed the formation of the bacterial and archaeal networks early in plant development and only inhibited nitrification at specific sampling times under field conditions. Sorgoleone was also shown to both inhibit and promote cultured bacterial isolate growth in laboratory tests. These findings provide new insights into the role of secondary metabolites in shaping the composition and function of the sorghum root-associated bacterial microbiomes. Understanding how root exudates modify soil microbiomes may potentially unlock an important tool for enhancing crop sustainability and yield in our changing environment.

**IMPORTANCE** Plant roots exude a complex mixture of metabolites into the rhizosphere. Primary and secondary metabolites exuded from roots are key drivers of root-soil microbe interactions that contribute to the structure and function of microbial communities in agricultural and natural ecosystems. Previous work on plant root exudates and their influence on soil microbes has mainly been restricted to model plant species. Plant are a diverse group of organisms and produce a wide array of different secondary metabolites. Therefore, it is important to go beyond studies of model plants to fully understand the diverse repertoire of root exudates in crop plant species that feed human populations. Extending studies to a wider array of root exudates will provide a more comprehensive understanding of how the roots of important food crops interact with highly diverse soil microbial communities. This will provide information that could lead to tailoring root exudates for the development of more beneficial plant-soil microbe interactions that will benefit agroecosystem productivity.

## INTRODUCTION

The rhizosphere is the layer of soil surrounding plant roots that is enriched with plant metabolites (e.g., secondary metabolites, carbohydrates, amino acids, organic acids, and hormones) (1). Due to the limited nutrients and carbon availability in the bulk soil (2), the rhizosphere becomes a relatively nutrient-rich microenvironment harboring microbes with diverse metabolic capacities. Advances in metabolomics and pioneering studies in exometabolomics (3, 4) have sparked renewed interest in studying how roots in plants such as *Arabidopsis* (5–7) and Avena barbata (8) influence the microbiome through the exudation of specialized compounds. The wide variety of compounds exuded from roots (9) selectively regulates the structure of microbial communities in the endosphere, rhizosphere, and bulk soil (10–13), which varies depending on plant species, stage of plant development, environmental conditions, and other factors (1, 8, 9, 14). While some work has been conducted using model plant species (5, 8) the exploration of how specialized secondary metabolites influence the composition and function of the rhizosphere microbiome is still in its infancy. Difficulties facing such studies include the vast array of secondary metabolites produced by plants and the lack of root exudate mutants or characterized natural variation available even in model systems such as *Arabidopsis*.

The effects of some secondary metabolites that are exuded from crop plant roots on soil microbe have been studied. 2,4-Dihydroxy-7-methoxy-1,4-benzoxazin-3-one (DIMBOA) is a major benzoxazinoid component of maize root exudates and one of the few root exudates in crop plants whose role in influencing the rhizosphere and root microbiomes has been extensively characterized. DIMBOA has multiple impacts on the root and rhizosphere microbiome, including attracting plant growth-promoting bacteria such as Pseudomonas putida to the rhizosphere and restructuring the composition of the microbiome (15–18). Flavonoids released to the soil by legumes (e.g., soybean) also act as chemoattractants that initiate *Rhizobium* plant root interactions leading to nodulation (19). Phenylpropanoids exuded from *Arabidopsis* roots attract the plant growth-promoting bacterium Pseudomonas putida to efficiently degrade many persistent organic pollutants (20). Additionally, plants also produce antimicrobials and other compounds to shape the microbiome and antagonize pathogens (21). Gaining comprehensive insight into the role of root-secreted compounds and how they shape the rhizosphere and endosphere microbiomes will provide new insights into the belowground interactions between plants and microbes and may also provide new approaches to enhance plant productivity through engineering metabolite exudation processes to positively influence the composition of the root associated microbiome.

Sorghum, the fifth most important cereal crop worldwide, exudes a unique class of lipophilic benzoquinones (sorgoleone) only from root hairs (22, 23) and not from other root tissues. Sorgoleone is the most well-studied hydrophobic component exuded from root hairs of sorghum, and some of its biological activities have been characterized (23). Its allelopathic properties (24) suppress the growth of surrounding plants (25) but can also cause self-toxicity for germinating seedlings (25). Sorgoleone also suppresses nitrification by blocking the activity of ammonia monooxygenase (AMO) and hydroxylamine oxidoreductase (HAO) in nitrifying bacteria. Nitrification mediated by soil bacteria and archaea is the conversion of ammonia or ammonium to nitrate. Under greenhouse conditions, biological nitrification inhibition (BNI) activity has been shown to increase nitrogen use efficiency (NUE) of sorghum and helps in adaptation to low-nitrogen soils (26, 27). The inhibition of nitrification by agricultural crop roots is important for reducing the conversion of ammonia to the more mobile form of nitrogen, i.e., nitrate. BNI, due to crop plant roots (27) such as sorghum, may reduce rapid nitrification, thereby reducing excess leaching of nitrogen below the root zone where it would become unavailable for plant uptake. It may also serve as a carbon source for soil microbes (28). However, field-based studies are needed to better understand the spatial and temporal effects of sorgoleone on BNI in agroecosystems and on microbial communities in soil.

The objectives of this study were to determine the impact of sorgoleone under field conditions on the composition of belowground microbial communities and to determine how sorgoleone influences nitrification. Wild-type sorghum was compared to transgenic sorghum events with large reductions in sorgoleone exudation (29). Field studies were conducted with these sorghum events to analyze the composition and network structure of microbial communities in the root, rhizosphere, and bulk soil using a 16S amplicon sequencing approach. In the field, sorgoleone altered the composition and network organization of microbial communities in the rhizosphere and in soil near sorghum roots through bacterial growth inhibition and promotion. The changes in the microbiome in the field were dependent on plant age or residence time in soil and did not have any obvious effects on plant development. The temporal effects of sorgoleone on soil BNI were assessed and showed time-dependent effects of sorgoleone on nitrification. Greenhouse trials with three levels of soil nitrogen revealed that soil nitrogen was important for sorgoleone-induced changes in rhizosphere and endosphere microbiomes. The direct effect of sorgoleone on bacteria was confirmed in the laboratory with cultured bacteria and purified sorgoleone.

## RESULTS

### Field census.

Two transgenic sorghum RNA interference (RNAi) events (pARS1-RNAi and pARS2-RNAi) that resulted in reduced sorgoleone production were previously described (29). The transcripts of Sobic.005G164300.1 and Sobic.008G036800 corresponding to *ARS1* and *ARS2* genes involved in sorgoleone biosynthesis were detected, with highest expression early in plant development at approximately 8 days after emergence of seedlings and then gradually decreasing from 24 days to 96 days (see Fig. S1d in the supplemental material) (30, 31). Low sorgoleone production (10 to 20 times less than the wild type) was confirmed in sorghum with these events by gas chromatography with flame ionization detection (GC-FID) analysis of the root exudates (Fig. S1a and b) and by light microscopy of the root hairs (Fig. S1c) in 1-week-old seedlings. Plant phenotypes collected during the 2016 field experiment showed no differences in biomass or chlorophyll content, but RNAi lines had decreased panicle weight (Fig. S1e).

10.1128/mSystems.00749-20.1 FIG S1 Sorgoleone in the root exudates of 1-week-old plants of the wild type, RNAi event no. 1, and RNAi event no. 2 lines. (a) Gas chromatography with flame ionization detection (GC-FID) analysis of dihydrosorgoleone in the root exudates of 1-week-old sorghum seedlings for the wild type and sorghum with RNAi events grown in the lab. RNAi no. 1 and RNAi no. 2 correspond to the events with the knockdown of the expression levels of ARS1 and ARS2 genes, respectively. The arrows on the top of the graph indicate the dihydrosorgoleone and their isoforms with a different number of double bonds at the side chain, which is thought to be a reduced form and is autooxidated once secreted into the rhizosphere soil to yield sorgoleone, a more stable benzoquinone. (b) Quantification of the dihydrosorgoleone (retention time = 9.6 min) for the wild type and RNAi events in the root exudates of 1-week-old seedlings. ***, *P* < 0.001 by one-way ANOVA analysis between wild-type and RNAi lines. (c) Light microscope observation of the root exudates at the end of the root hair of the wild-type and RNAi event-harboring sorghum. The red arrowheads indicate the exudate droplets containing sorgoleone secreted from the root hair tips. (d) Expression level patterns of ARS1 and ARS2 in the root bottom of sorghum BTx623 using the data obtained from the Phytozome database. (e) Dry panicle weight from the main stem of wild-type and RNAi event-harboring sorghum samples that were harvested from a field in 2016 (*N* = 5 to 7). Panicle weights with different lowercase letters indicate significant differences in the panicle weight between genotypes at α = 0.05 by Tukey honestly significant difference (HSD) multiple-comparison test. Download FIG S1, TIF file, 0.7 MB. Copyright © 2021 Wang et al. 2021 Wang et al. https://creativecommons.org/licenses/by/4.0/ This content is distributed under the terms of the Creative Commons Attribution 4.0 International license.

The β-diversities of the microbial communities were different at the three different sampling times in endosphere, rhizosphere, and soil near roots (see Fig. S2). Soil samples proximal to the rhizosphere instead of the bulk soil were collected to explore the effect of sorgoleone on the soil just outside the rhizosphere. These samples are referred to as “soil near roots.” In addition, some samples were taken from soil between the rows of sorghum, and those are referred to as “soil between rows.”

10.1128/mSystems.00749-20.2 FIG S2 β-Diversity analysis for endosphere, rhizosphere, and soil near roots from the 2016 field trial. Canonical analysis of principal coordinates (CAP) was performed on Bray-Curtis distance matrix constrained by sampling time and factoring out genotype. The *P* value and *R*^2^ on the left top indicate the constrained PERMANOVA results. Download FIG S2, TIF file, 0.3 MB. Copyright © 2021 Wang et al. 2021 Wang et al. https://creativecommons.org/licenses/by/4.0/ This content is distributed under the terms of the Creative Commons Attribution 4.0 International license.

A constrained permutational multivariate analysis of variance (PERMANOVA) (see Table S1) testing the influence of plant stage on microbial community composition (factoring out genotype) showed that sampling time or plant development had a highly significant effect on the microbial community composition (*P* = 0.001). In the rhizosphere, sampling time contributed to approximately 23% of variation in the composition of microbial communities, 17% of the variation in the soil near roots, and 11% in the endosphere.

10.1128/mSystems.00749-20.5 TABLE S1 Parameters used in statistical analyses of beta diversity analysis for rhizosphere, endosphere, and soil samples from 2016 field and greenhouse experiments. Partial canonical analysis of principle coordinates (CAP) was performed on Bray-Curtis distance metric according to different models to understand the quantitative impact of the constrained factors of interest on the bacterial composition and, at the same time, controlling for the other factors to factor out their effects. Download Table S1, XLSX file, 0.01 MB. Copyright © 2021 Wang et al. 2021 Wang et al. https://creativecommons.org/licenses/by/4.0/ This content is distributed under the terms of the Creative Commons Attribution 4.0 International license.

**FIG 1:**
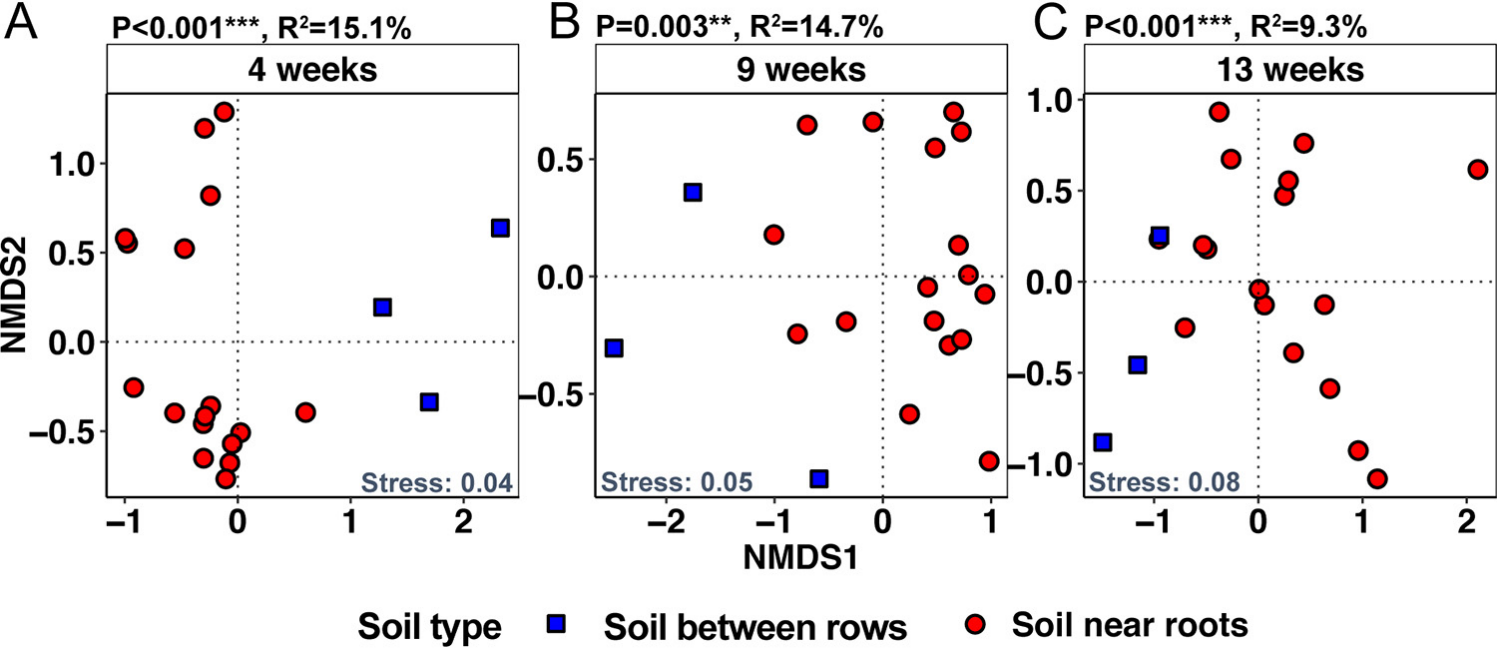
Soil bacterial β-diversity/community composition in the field soil between rows and the soil near sorghum roots at three time points during the field season in 2016. Four weeks corresponds to the seventh-leaf stage, 9 weeks to boot stage, and 13 weeks to grain fill stage. All the soil samples separated by time for NMDS ordination by using the Bray-Curtis matrix. The *P* values and *R*^2^ on the top left of each graph show the PERMANOVA results. The stress values at the bottom right corners of the plots reflect how well the ordination summarizes the observed distances among the samples.

Differences in microbial community composition between soil sampled between rows and soil near roots were assessed by nonmetric multidimensional scaling (NMDS) analysis at each sampling time (Fig. 1). A constrained PERMANOVA revealed a highly significant difference between the soil near roots and soil between rows at all three sampling times (Fig. 1). The sampling time had no effect on the soil-between-rows microbial communities (PERMANOVA *P* = 0.11) (Table S1d).

**FIG 2:**
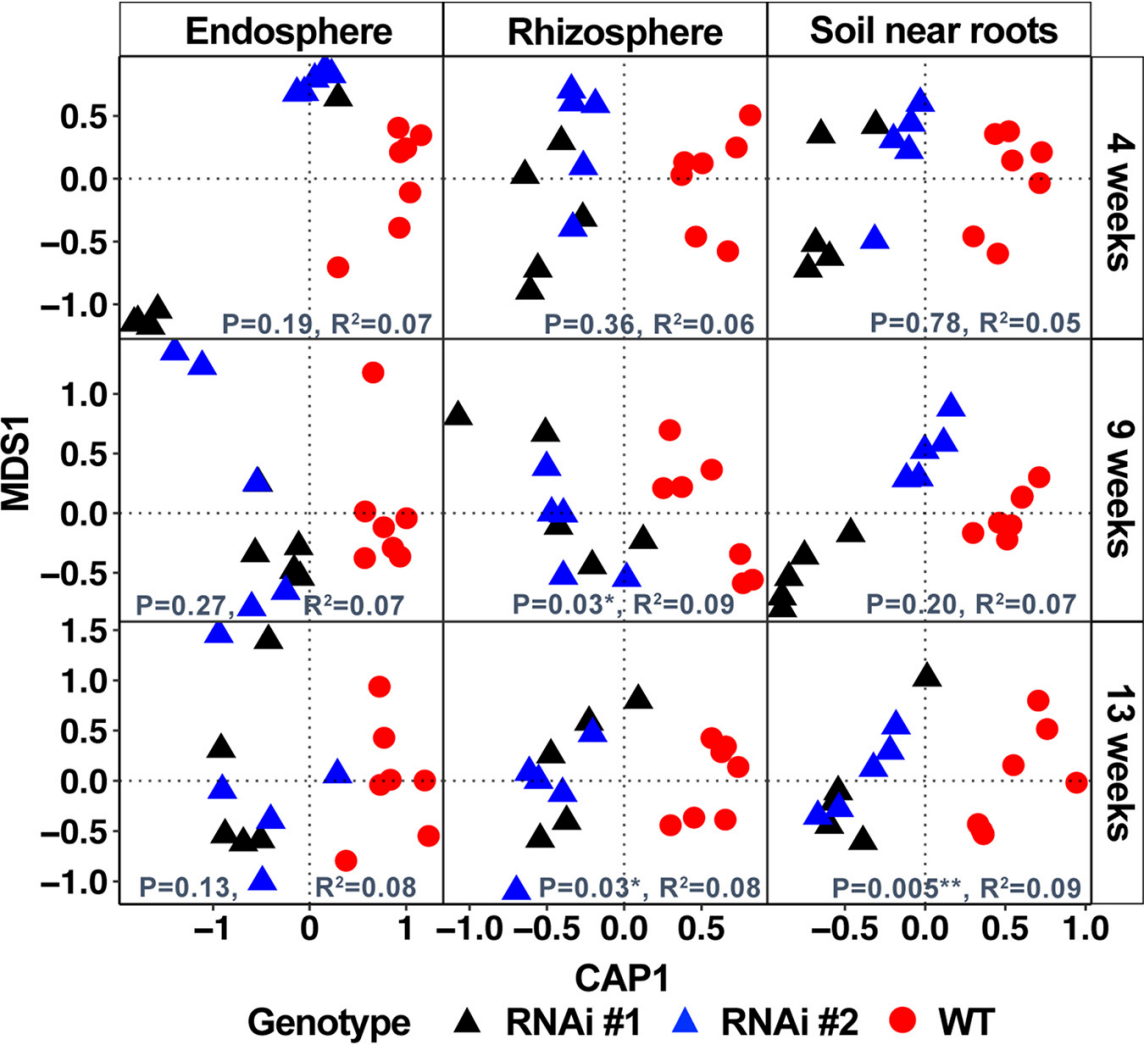
Canonical analysis of principal components (CAP) of bacterial and archaeal community composition in the endosphere, rhizosphere, and soil near roots of two RNAi event-harboring and wild-type sorghum at three times during the field season in 2016. The CAP was constructed using the Bray-Curtis dissimilarity matrix constrained to the genotype (sorghum with RNAi events and wild type). The PERMANOVA results are shown along the *x* axis of each plot. Triangles represent the samples from the sorghum with RNAi event (*n* = 5 for each) and circles represent the wild-type samples (*n* = 7). Four weeks, 9 weeks, and 13 weeks correspond to the seventh-leaf, boot, and grain fill stages, respectively.

Since sampling date had a strong influence on shaping the bacterial communities (Fig. S2), samples were separated into individual time points to better understand the temporal influence of sorgoleone on microbial communities. Canonical analysis of principal coordinates (CAP) was used with genotype being the primary variable tested. There were differences between sorghum with RNAi events as compared to the wild type (WT) in the composition of the microbial communities in the rhizosphere at the two later stages of development (boot and grain fill stage) (*P* = 0.03) and that of the microbial communities in the soil near roots (*P* = 0.005) at the last sampling at week 13 (Fig. 2; Table S1a and c). There were no significant differences in endosphere microbial communities (Fig. 2; Table S1b). The total variation due to sorgoleone exudation was between 8% and 9% in the rhizosphere and soil near root. Most soil chemical properties were similar between the plots where the sorghum with RNAi events and wild-type sorghum were grown (see Table S6) except when analyzed based on individual sampling dates, where the organic matter in the soil near roots was significantly higher in the wild type at the earliest sampling at week four (see Fig. S3a).

10.1128/mSystems.00749-20.3 FIG S3 Soil organic matter data at each sampling date from the 2016 field experiment and relative abundance of two OTUs that were both stimulated in laboratory and field data. (a) Soil organic matter measurements in the soil near roots from the 2016 field experiment. (*n* = 7 for wild type; *n*= 5 for each of RNAi number 1 and RNAi number 2; *n* = 3 for soil between rows). The means ± standard errors (SEs) are shown. *, *P* < 0.05 by Student’s *t* test between wild type and RNAi lines. (b) Relative abundance of two OTUs, Otu152 and Otu521, from data of rhizosphere collected in 2015 and 2016 field and greenhouse experiments at the different sampling times. Otu152 and Otu521 are the two OTUs that are assigned to *Methylophilaceae* at the family level and *Nocardia* at the genus level, respectively. These OTUs were in significantly higher abundance in the wild type than in the RNAi lines in the differential abundance analysis using DESeq2 and also showed a positive response to the sorgoleone in laboratory assays. *, *P < *0.05; **, *P < *0.01; ***, *P < *0.001 between wild-type and RNAi lines using the Wilcoxon nonparametric statistical test. Download FIG S3, PDF file, 0.27 MB. Copyright © 2021 Wang et al. 2021 Wang et al. https://creativecommons.org/licenses/by/4.0/ This content is distributed under the terms of the Creative Commons Attribution 4.0 International license.

10.1128/mSystems.00749-20.10 TABLE S6 Soil chemical analysis from Mead, NE, 2016 field trials. Values in table are averages from each mineral in soil near roots (*n* = 17) and soil between rows (*n* = 3) at each sampling time. The different lowercase letters indicate a significant difference between soil near roots and bare soil at different sampling times at α = 0.05 by Tukey HSD multiple-comparison test. Download Table S6, XLSX file, 0.01 MB. Copyright © 2021 Wang et al. 2021 Wang et al. https://creativecommons.org/licenses/by/4.0/ This content is distributed under the terms of the Creative Commons Attribution 4.0 International license.

### Sorgoleone inhibition assay.

**FIG 3:**
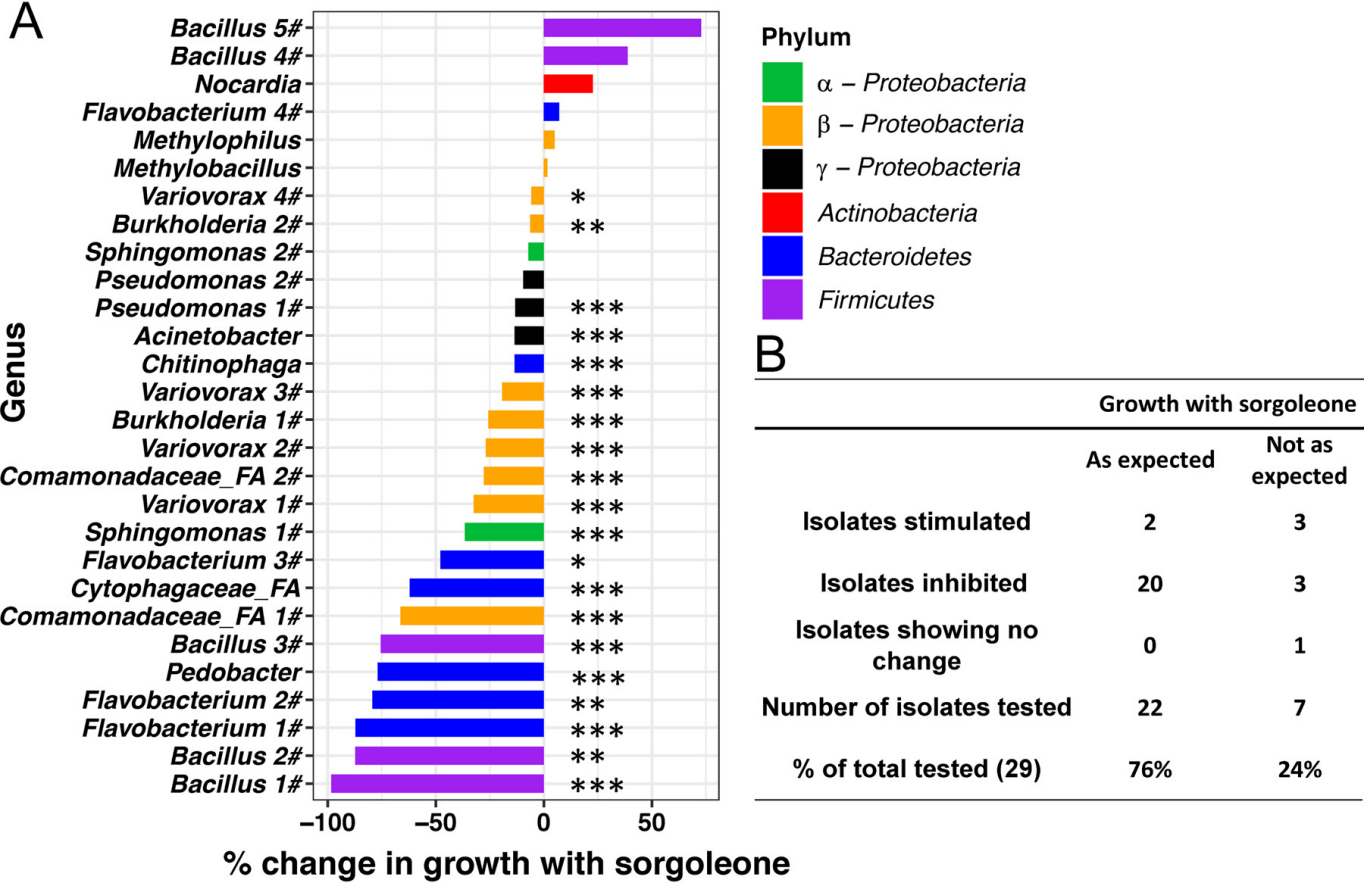
Growth of bacterial isolates with sorgoleone. (A) Isolates were selected from a culture collection based on genus- or family-level similarity using differential abundance data from wild type and sorghum with RNAi event endosphere and rhizosphere data from field studies (summarized in Table S2 in the supplemental material). Percentage inhibition by 0.025 mM sorgoleone was calculated by [(OD_without sorgoleone_/OD_with sorgoleone_) × 100] − 100. Welch’s *t* test was used due to unequal variance between sorgoleone-treated samples and control samples. *P* values from one-sided tests were converted to *q* values to control for false-discovery rates. ***, *P* < 0.001; **, *P*, < 0.01; *, *P* < 0.05. (B) Table indicates how the *in vitro* results matched with culture-independent data for the 29 isolates (Table S2). α-Proteobacteria, *Alphaproteobacteria*; β-Proteobacteria, *Betaproteobacteria*; γ-Proteobacteria, *Gammaproteobacteria*.

The operational taxonomic units (OTUs) that differed in abundance between the WT and sorghum with RNAi events were identified using a negative binomial model in DESeq2 and a false-discovery rate (FDR) adjusted *P* value (*P_adj_*) of <0.05 for the 2015 and 2016 and greenhouse data sets (see Table S2). Cultured isolates that matched the genus or family of differentially abundant microbes (Table S2 and Table S3) were used for *in vitro* growth studies that confirmed sorgoleone had the potential to inhibit certain soil microbial taxa in the laboratory and also enhance the growth of other soil microbes (Fig. 3). A sorgoleone-sensitive Nitrosomonas europaea strain (ATCC strain 19718) transformed with a LUX marker was used in a luminescence assay as a control (26, 32). The expected inhibition of the luminescence by sorgoleone was observed. Approximately 76% of the isolates responded to sorgoleone, as was expected based on the culture-independent 16S data analysis, and it is interesting to note that most (23/29) isolates tested were inhibited by sorgoleone (Fig. 3B). The growth of one *Methylobacillus* isolate showed little response to the sorgoleone in the assay (−2.9%). Growth of five isolates was stimulated by sorgoleone. Two of the isolates in the laboratory assays, *Methylophilus* and *Nocardia*, whose growth was stimulated by sorgoleone were also consistently higher in relative abundance in the rhizosphere of the wild-type sorghum than in that of sorghum with the RNAi events in 2015 and 2016 field and greenhouse experiments (Fig. S3b). Five *Bacillus* isolates were randomly chosen from our culture collection, and the growth of three were strongly inhibited by sorgoleone, while two were stimulated. The three inhibited were classified as B. safensis and B. cereus while the two that were stimulated were classified as B. flexus (Table S3).

10.1128/mSystems.00749-20.6 TABLE S2 Differential abundance analysis of OTUs to determine which ones are significantly more or less abundant due to the presence or absence of sorgoleone. During the analysis, RNAi no. 1 and RNAi no. 2 data were combined, as the “absence of sorgoleone” group to compare to the wild type as the “presence of sorgoleone” group. Only the OTUs showing significantly different absolute abundance between wild-type and RNAi lines at an adjusted *P* value (*P*_adj_) of ≤0.05 are listed in this table. The negative value of log2 fold change indicates the OTUs are more abundant in RNAi lines, and the positive value of log2 fold change indicates the OTUs are more abundant in WT. Download Table S2, XLSX file, 0.03 MB. Copyright © 2021 Wang et al. 2021 Wang et al. https://creativecommons.org/licenses/by/4.0/ This content is distributed under the terms of the Creative Commons Attribution 4.0 International license.

10.1128/mSystems.00749-20.7 TABLE S3 Response to the addition of sorgoleone to growth medium for bacterial isolates chosen based on the analysis shown in Table S2. The isolate identifier (ID) in the lab database, the source of these tested isolates, the taxonomic information, the predicted response to the sorgoleone for each isolate based on culture-independent data, the corresponding OTUs and their statistical analysis in the differential abundance analysis, and the inhibition due to added sorgoleone in the growth medium are shown. The taxonomic information of these isolates was annotated based on the 1.5-kb length of 16S rRNA sequences from Sanger sequencing. A sorgoleone concentration of 0.025 mM was added to each well. Download Table S3, XLSX file, 0.02 MB. Copyright © 2021 Wang et al. 2021 Wang et al. https://creativecommons.org/licenses/by/4.0/ This content is distributed under the terms of the Creative Commons Attribution 4.0 International license.

### Nitrification potential.

**FIG 4:**
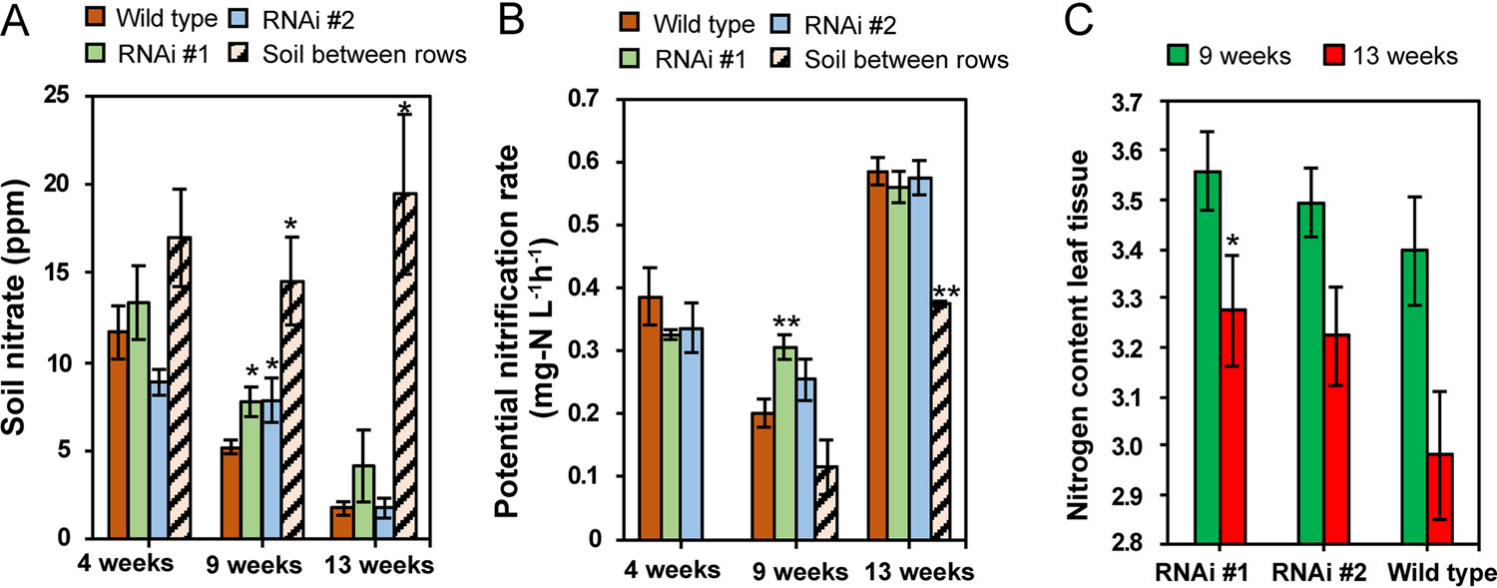
Soil nitrate concentration, potential nitrification rates of soil near roots, and nitrogen content in leaves of wild-type and sorghum with RNAi events in the 2016 field season. (A) Soil nitrate content. (B) Potential nitrification rates. The soil-between-rows sample data at 4 weeks was missing. (C) Percent nitrogen content in leaf tissue of WT and RNAi event-harboring sorghum. **, *P* < 0.01; *, *P* < 0.05 between wild-type and RNAi or soil between rows using Student’s *t* test.

Sorgoleone inhibits certain taxa of nitrifying bacteria (26, 33). To test for changes in nitrification due to sorgoleone across a growing season under field conditions, soil near roots was examined carefully at three sampling times. Sorgoleone inhibited nitrification in field soil at the 9-week sampling of the soil near roots (Fig. 4A and B). The soil nitrate concentrations (Fig. 4A) and potential nitrification rates (Fig. 4B) were higher in the near soil of the sorghum with RNAi events than in near soil where the wild type was grown. Potential nitrification rates could not be measured in the rhizosphere because of the small amount of soil recovered from the rhizosphere isolation. Leaf nitrogen content was higher in sorghum with both RNAi events than in wild type sorghum at weeks 9 and 13, but only that with RNAi event number [no.] 1 had significantly higher (*P* < 0.05) levels of nitrogen at 13 weeks (Fig. 4C). The soil-between-rows nitrate concentrations were relatively constant throughout the season. In contrast, nitrate concentration was progressively depleted in soil near roots across the season, presumably due to plant nitrogen uptake (Fig. 4A).

### Greenhouse experiment.

**FIG 5:**
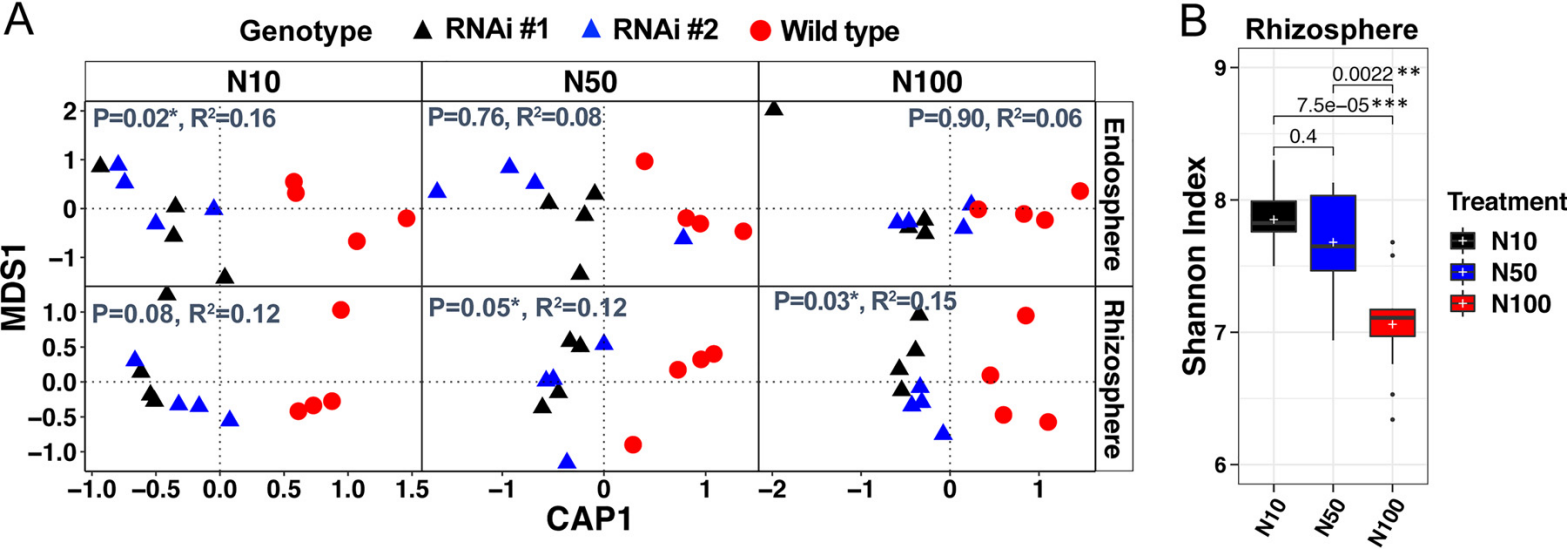
β-Diversity analysis for rhizosphere, endosphere, and soil samples from a greenhouse experiment conducted with different nitrogen levels (N10, 1.15 mM N; N50, 5.75 mM N; N100, 11.5 mM N). (A) Canonical analysis of principal coordinates (CAP) was performed on Bray-Curtis distance matrix in which treatment was constrained and genotype was factored out. The *P* values and *R*^2^ on the top left of each graph show the PERMANOVA results. (B) Shannon index for rhizosphere under nitrogen treatment conditions in the greenhouse. The *P* values shown on the top were calculated by the Wilcoxon nonparametric statistical test. Line and the plus marker in the boxes represent median and mean, respectively, top and bottom of boxes represent first and third quartiles, respectively, and whiskers indicate 1.5 interquartile range.

Under greenhouse conditions, soil nitrogen content altered how sorgoleone influenced the microbial communities (Fig. 5). The endosphere bacterial communities differed (PERMANOVA *P* = 0.02) between WT and sorghum with RNAi events only at the lowest soil nitrogen content. In contrast, differences in microbial community composition in the rhizosphere between wild type and RNAi events were significant at the two highest levels of nitrogen (*P* = 0.05 at 5.75 mM N [N50] and *P* = 0.03 at 11.5 mM added N [N100]) (Fig. 5A). In rhizosphere, the α-diversity decreased as nitrogen increased from 1.15 mM added N (N10) to the N100 level in both WT and RNAi events (Fig. 5B) but was not statistically different between RNAi events and WT at all nitrogen treatments (see Table S4).

10.1128/mSystems.00749-20.8 TABLE S4 α-Diversity analysis showing the Shannon index for sorgoleone effect in 2015 and 2016 field and greenhouse experiments. **, *P* < 0.01 by using the Wilcoxon nonparametric statistical test. Download Table S4, XLSX file, 0.01 MB. Copyright © 2021 Wang et al. 2021 Wang et al. https://creativecommons.org/licenses/by/4.0/ This content is distributed under the terms of the Creative Commons Attribution 4.0 International license.

### Co-occurrence networks.

**FIG 6:**
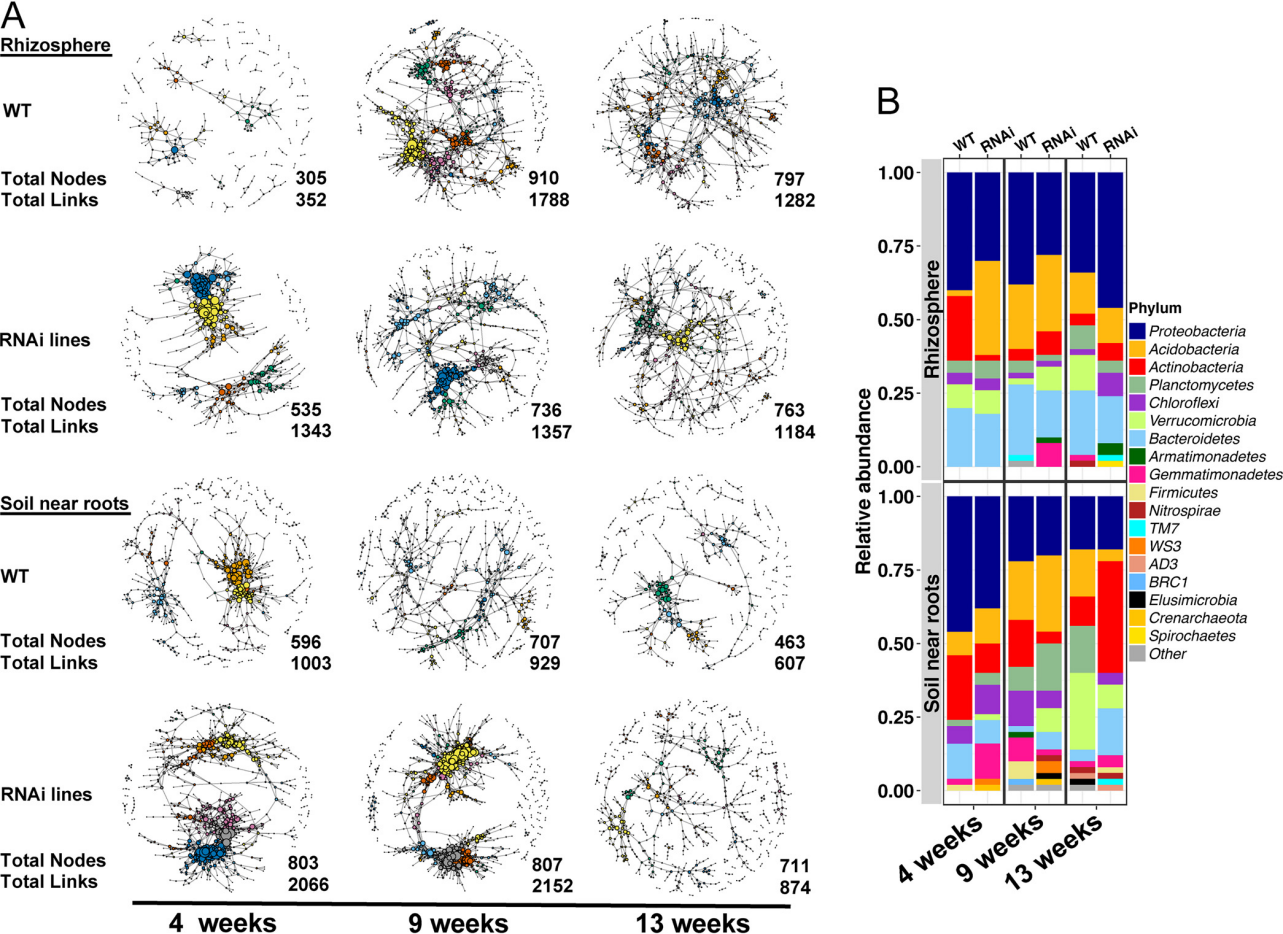
Co-occurrence OTU network analysis and relative abundance of the 50 OTUs with the highest hub scores. (A) Bacterial and archaeal co-occurrence networks over time as affected by sorghum genotype (wild type or RNAi events) and sample type in 2016 field. Node represents individual OTU; edges represent significant positive Spearman correlations (ρ > 0.8 and *P* value ≤0.01). Number of the total nodes and links shown at the bottom right of each network. (B) The relative abundance of the 50 OTUs with the highest hub score in each network is displayed. The *x* axis indicates the time and genotype, with the relative abundance shown on the *y* axis. Top graph indicates the rhizosphere and bottom indicates the soil near roots. Color of bar indicates the different phyla.

The reduction of sorgoleone in root exudates of sorghum with the RNAi events tended to increase the bacterial and archaeal network complexity in the rhizosphere and in the soil near the roots early in plant development compared with that for the wild type (Fig. 6A and Table S5). The total nodes and total links (connections) between nodes were higher in the rhizosphere for the sorghum with RNAi events at week four and throughout the season for the soil near roots, as indicated by larger network size with more nodes and a more connected network. The three-way analysis of covariance (ANCOVA) confirmed differences between the RNAi events and the wild type, with a higher normalized node degree (number of node connections was normalized by the total number of nodes) for the RNAi events when the data from all time points and both rhizosphere and soil near roots were considered together (*F*_1,8121_ = 42.2, *P* < 0.001) (see Fig. S4a). There were differences in the normalized node degree at 4 weeks for rhizosphere and 9 weeks and 13 weeks in the soil near roots (Fig. S4a). Betweenness (the extent to which a node is connected to other nodes that are not connected to each other) of the networks also was different for sorghum with RNAi events and the wild type. At week four in rhizosphere and soil near roots, the betweenness was higher (Fig. S4b) for the RNAi events than for the wild type. There were also differences in closeness (a measure of the degree to which a node is near all other nodes in a network) of nodes between the wild type and the sorghum with RNAi events in the rhizosphere throughout the experiment (Fig. S4c). More closeness of the networks in sorghum with RNAi events was observed compared to the wild type at week four in the soil near roots. Time was also a significant factor in changes in the network characteristics such as module number (ANCOVA statistics in Table S5), normalized node degree (Fig. S4a), closeness centrality (Fig. S4c), and in betweenness centrality (Fig. S4b). Taken together, the results of the network analysis highlight the changes in connectivity and network size due to the root exudate sorgoleone.

10.1128/mSystems.00749-20.4 FIG S4 Node connectedness (normalized degree) (a), betweenness centrality (b), and closeness centrality (c) of bacterial and archaeal networks in the rhizosphere and soil near roots from 2016 of the wild-type and the RNAi lines at each sampling time. Dots represent single observations from OTUs. The white line in the boxes indicates median, top and bottoms of boxes represent first and third quartiles, respectively, and whiskers indicate 1.5 interquartile range. Different lowercase letters indicate a significant difference of normalized degree between and within genotypes at α = 0.05 by Tukey HSD multiple-comparison test. The F tables show the three-way ANCOVA of the interaction between sample type, time, and genotype of the normalized node degree in the network across the three sampling times in all the sample types. Download FIG S4, TIF file, 0.5 MB. Copyright © 2021 Wang et al. 2021 Wang et al. https://creativecommons.org/licenses/by/4.0/ This content is distributed under the terms of the Creative Commons Attribution 4.0 International license.

10.1128/mSystems.00749-20.9 TABLE S5 Topological properties and ANCOVA of the empirical co-occurrence networks of the rhizosphere and soil near roots. The networks were separated by genotype at different plant growth stages. Download Table S5, XLSX file, 0.07 MB. Copyright © 2021 Wang et al. 2021 Wang et al. https://creativecommons.org/licenses/by/4.0/ This content is distributed under the terms of the Creative Commons Attribution 4.0 International license.

The 50 OTUs with the highest hub scores (34) were visualized at the three sampling times (Fig. 6B). There was a higher relative abundance of *Actinobacteria* and *Proteobacteria* in both the wild-type rhizosphere and soil near roots 4 weeks after planting than at other sampling times, whereas the sorghum with RNAi events had greater relative abundance of *Acidobacteria* at that same time point. At week nine and later sampling times, the relative abundances and the network maps in the soil near roots began to look more similar between the wild type and the sorghum with RNAi events. The differences in relative abundance of these key OTUs became smaller as the bacterial community matured later in the season, except at 13 weeks, when the wild type contained a much higher relative abundance of both *Verrucomicrobia* and *Planctomycetes* in the rhizosphere and soil near roots, while a higher abundance of *Actinobacteria* was observed in the sorghum with RNAi events in the soil near roots.

## DISCUSSION

Root exudates are an important emerging area of plant and soil science research (9, 35–37). While the role of relatively few secondary metabolites in root exudates has been clearly defined, some have been shown to be key drivers influencing microbial community composition within the rhizosphere (5, 6, 15, 16, 18, 38–40). Sorgoleone is the major secondary metabolite in Sorghum bicolor root exudates (29). This lipid benzoquinone has a number of biological activities, but much attention has centered on the allelopathic aspects of this root exudate (23). More recently, it was shown that sorgoleone inhibits soil-nitrifying bacteria mainly in greenhouse experiments or in lab assays (26, 33). Since sorghum is an important worldwide cereal crop and is also rapidly becoming an important bioenergy crop (41), this study focused on determining whether sorgoleone alters the bacterial and archaeal communities of the root microbiome and whether it influences the nitrogen cycling under field conditions.

### Sorgoleone alters composition of bacterial microbiome.

Several recent agricultural fields studies reported that crops influence their soil microbiomes dynamically across a growing season (42, 43). Natural variation exists in the amount of sorgoleone exuded from sorghum root hairs of different sorghum lines (44), but no reports have shown the impact of this root exudate on the rhizosphere, endosphere, or soil microbiome composition under field conditions. Although the impacts of sorgoleone on the soil microbiome were recently shown under greenhouse conditions (45), such approaches are notoriously difficult to translate to field conditions due to the restricted soil volumes and higher soil temperatures. Therefore, our work provides a novel and relevant data set for studying the role of sorgoleone on rhizosphere and endosphere bacterial and archaeal communities in agricultural systems. The availability of sorghum that carries RNAi elements designed to downregulate two specific type III polyketide synthases (PKSs) (29) involved in the synthesis of sorgoleone permitted the direct testing of how this exudate influenced the composition and function of the soil bacterial microbiome in isogenic genetic backgrounds. The results of these studies showed that the presence of large amounts of sorgoleone shifts the composition of the bacterial and archaeal microbiome. Although previous work (26, 33) has shown that specific nitrifying bacteria are inhibited by sorgoleone, our findings revealed that the abundance and growth of a much wider range of bacterial taxa are both inhibited and promoted by sorgoleone in the rhizosphere. In follow-up investigations, experimental protocols ensuring a higher sequencing depth for root samples will be required to further elaborate on the conclusions related to the endosphere microbiome reached in this paper. These findings are supported by results from field studies, a greenhouse study, and laboratory growth assays on cultured bacterial isolates.

The greenhouse experiments suggested that sorgoleone plays a role in altering the rhizosphere bacterial microbiome only when sufficient amounts of nitrogen are present. These experiments also highlight changes in endosphere microbial communities that may occur only under very low nitrogen conditions. Although the conditions under which microbial nitrification inhibition is important in agricultural fields are not well understood (46), it is known that inhibiting nitrate leaching by the addition of chemical nitrification inhibitors is one approach for reducing nitrogen losses (47). The presence of adequate nitrogen would likely be required for the activity of nitrifying bacteria, because ammonia is the major source of energy for both bacteria and archaea involved in nitrification (46). In addition, the amount of certain phenolics and lipids in exudates increases with higher nitrogen concentration (48), suggesting that the amount of root exudation may be dependent on the nitrogen level. Therefore, higher levels of nitrogen may lead to greater exudation of sorgoleone, leading to significant changes in bacterial community composition in the rhizosphere. The greenhouse results confirmed trends measured in the field, where higher concentrations of sorgoleone exuded from wild-type sorghum roots led to alterations in the composition of the rhizosphere bacterial microbiome.

### Network analysis.

In addition to the use of β-diversity to characterize changes in microbial communities, co-occurrence network analysis has emerged as an important approach for revealing information on the co-oscillation of community members and the stability of community assembly (49–51). In previous work, co-occurrence network analysis of the microbial communities highlighted the increased connectivity and complexity of bacterial assemblages in rhizosphere compared to those in bulk soil (50). While our results showed similar network complexity in the rhizosphere, the soil near roots (just outside the rhizosphere) also had complex networks, which suggests it was influenced by the root exudates, including sorgoleone. Sampling further away from the roots between the rows of sorghum showed no change over time, again suggesting the lack of influence of root exudation. However, it is not clear why the network organization of the soil near roots was more complex than that in the rhizosphere at the early sampling. One possibility for this difference may be due to moisture gradients between the rhizosphere and soil near roots. While the soil moisture of the rhizosphere was not measured, a gradient was observed between the wetter soil between rows and the drier soil near roots (see Table S6 in the supplemental material), which was likely due to plant uptake of soil water. Therefore, assuming that the soil was drier in the rhizosphere, this may have reduced microbial activity, leading to less complex networks early in development than that in the soil near the roots (52).

The delayed formation of the complex microbial co-occurrence networks observed in the wild-type sorghum early in plant development was ascribed to the presence of larger amounts of sorgoleone that may be produced earlier in plant development based on the transcriptional profiles of the ARS genes involved in sorgoleone biosynthesis (Fig. S1d). The total nodes and total links (connections) between nodes were higher in the rhizosphere at week four and throughout the season for the soil near sorghum roots harboring the RNAi events (Fig. 6), indicating that the reduction of sorgoleone leads to larger and more connected bacterial networks. Others have also shown that environmental factors such as elevated CO_2_ (53) and agricultural intensification (54) lead to changes in the network organization of microbiomes. Inspection of the 50 OTUs with the highest hub scores showed that the phylum *Actinobacteria* was in greater relative abundance at week four in the wild type. One hypothesis is that sorgoleone imposes a “stress-like” effect on the microbial community (42) that delays formation of highly connected microbial networks. This work shows that changes in the microenvironment around the roots due to the exudation of plant secondary metabolites profoundly change microbial networks in soil.

Another possible reason for the changes in the early microbial communities may be due to the different roles that sorgoleone plays in the rhizosphere and the soil near roots in the creation of new niches for specific bacterial taxa (6, 55). Sorgoleone may change the competition between microbes, leading to changes in community composition or the creation of new niches for certain microbes to thrive. New metabolic niches that may be created by sorgoleone are likely to be due to its multiple roles as an inhibitor and growth promoter as confirmed by laboratory assays and as an inhibitor of specific enzymes in nitrifying bacteria (26).

Data from field and greenhouse experiments showed that two isolates from the genus *Nocardia* and the family *Methylophilaceae* were identified as microbes whose relative abundance was higher in the wild type (Fig. S3b). The growth promotion of *Nocardia* and *Methylobacillus* isolates was also observed in laboratory assays using cultured isolates. *Nocardia* species belonging to the phylum *Actinobacteria* have been shown to degrade complex organic compounds and may use sorgoleone as a carbon source for enhanced growth (56). Based on the demonstrated mineralization of sorgoleone in soil (28) and the laboratory assays in this study, it is likely that sorgoleone is being used as a carbon source for certain taxa with the metabolic capacity to utilize this unique lipid. The increase in the relative abundance of *Nocardia* was also correlated with higher soil organic matter (Fig. S3a) in the wild-type sorghum, which may be a cause or an effect of the increased abundance of *Nocardia*, which is also known for its ability to degrade lignin (57). The increase in the abundance of bacteria from the *Methylophilaceae* family was also noted in another study on the exudation of the secondary metabolite DIMBOA (18). Similar to DIMBOA, sorgoleone has a growth-inhibiting effect which may create a new niche for *Methylophilaceae* bacteria to thrive. Sorgoleone exuded from roots may also cause certain defense responses in sorghum due to self-toxicity (25), which may lead to the production of plant-derived methanol coming from pectin methylesterase activity that may be produced in cell walls when plant defense responses are triggered (18). Another possible explanation for the increased abundance of members of the *Methylophilaceae* family in WT is that the methoxy group of sorgoleone is released and becomes an energy source for these methylotrophs (28). Many *Nocardia* species are also known to produce antimicrobial compounds to suppress other microbes, resulting in a sparse network (56). Metabolites from both root exudates and from soil microbes lead to the creation of new niches for members of the microbial community through multiple mechanisms (6, 8, 55). The laboratory assays using cultured isolates provided supporting evidence for a growth-promoting role of sorgoleone that leads to a new hypothesis that sorgoleone creates specialized metabolic niches for bacteria and archaea through mechanisms of inhibition, niche creation, and direct growth promotion.

The changes observed in the rhizosphere and soil-near-root network confirmed the dynamic nature of the bacterial microbiome and suggested that sorgoleone begins to impact microbial relationships early in the season. However, changes in the bacterial microbiome β-diversity were significant only later in the season. Such timing differences in the two analyses suggest that the early changes in the networks, which may be due to higher levels of sorgoleone production early in development, lead to the observed differences in microbial community composition later in the growing season (58). Together these two types of analyses provide a more integrated understanding of the dynamics of soil microbial community structure in response to sorgoleone.

### Sorgoleone effect on soil nitrification.

Nitrification is an important process in the nitrogen cycle of agroecosystems, particularly in the highly productive areas of the United States where >90 million acres of maize are planted. In these systems, ammonia fertilizer is applied and either remains in the soil or is converted to nitrate that is readily leached into the ground and surface waters (59). The process of nitrification is due to nitrifying bacteria in the soil that convert ammonia to nitrate (60). While others have highlighted the potential for the use of sorgoleone exudates by sorghum as a potential nitrification inhibitor to reduce the loss of nitrogen from agroecosystems (26, 33, 45, 61), our data suggest that at only certain times during the season was nitrification inhibited by sorgoleone. Therefore, the overall extent of the impact of sorgoleone exudation on nitrification in field soils remains unclear. Two observations in this study suggest that biological nitrification inhibition through sorgoleone may be of limited importance for reducing the nitrate leaching from the soil. First, a reduction in nitrification was only detected after 9 weeks, similar to what was previously found (45). Second, the changes in the microbial community composition due to sorgoleone were only detected in the soils near roots (2016) and not in bulk soils. This suggests that the sorgoleone may not move far from the roots and therefore may have a limited impact on overall nitrification at the field level. The lack of movement of sorgoleone may be due to its high lipophilicity, which would prevent it from moving with soil water any distance from the point of exudation. While sorgoleone does inhibit nitrification at certain times during the season, the full effects of the RNAi events may be masked by other compounds that are exuded from sorghum roots such as sakuranetin (26, 59). Additional experiments will be required to fully assess whether sorgoleone inhibits nitrification to the extent that it could reduce nitrate leaching into ground water in agroecosystems. The use of plants to reduce nitrification has been discussed (33) and tested mainly in the greenhouse, with no larger-scale field-based experiments reported to our knowledge. Future field-based experiments that measure nitrate leaching and a full nitrogen balance will be required to fully assess the potential for sorgoleone as a nitrification inhibitor.

### Conclusions.

Taken together, the field-based and greenhouse experiments as well as laboratory-based growth assays with sorgoleone clearly demonstrate the role of this sorghum root exudate not only in inhibiting the growth and function of a wide range of different bacterial taxa but also in stimulating the growth of certain taxa. The work also highlights the important role of exudation of the secondary metabolite sorgoleone in shaping rhizosphere and soil microbial communities and possibly providing a unique metabolic niche for specific taxa to thrive in the rhizosphere and soil near roots. Consistent with previous observations, sorgoleone in root exudates impacts the nitrogen cycling transiently where it inhibits nitrification in the zone around roots. These findings provide additional insight into how altering these root exudation processes in plants may provide effective approaches for engineering soil microbiomes that enhance the stress tolerance and increase the productivity of agroecosystems.

## MATERIALS AND METHODS

### Plant material.

Two RNAi events were induced by transforming the immature embryos of Sorghum bicolor (genotype Tx430) with the constructs pARS1-RNAi (RNAi event no. 1) and pARS2-RNAi (RNAi event no. 2), and their corresponding wild-type Tx430 was also used in this study. These two RNAi events were induced by knocking down two polyketide synthase (PKS)-like genes encoding the ARS enzymes (ARS1 and ARS2) in the biosynthesis pathway of sorgoleone (29, 62). We confirmed the reduction of sorgoleone in the sorghum root hairs with the knockdown events by light microscope observation and GC-FID analysis (see Fig. S1a to c in the supplemental material). Whole sorghum root systems of 1-week-old plants were dipped in 15 ml of methylene chloride to collect exudate. Roots were then blotted dry and weighed. The exudate samples were then dried using nitrogen gas. Before loading to GC-FID, pyridine and *N*,*O*-bis(trimethylsilyl)trifluoroacetamide (BSTFA) plus trimethylchlorosilane (TMCS) (99:1) were used for the derivatization. GC-FID (Agilent) with HP-INNOWax polyethylene glycol was used. Inlet temperature was set at 250°C with a hydrogen flow rate of 37.2 ml min^−1^ at 25 lb/in^2^. The ramp protocol was 185°C for 1 min and then up to 240°C at a rate of 7°C min^−1^, followed by a hold at 240°C for 13 min. The temperature of the flame ionization detector was 275°C with a hydrogen flow rate of 45.0 ml min^−1^ and airflow rate of 375 ml min^−1^. In addition, fragments per kilobase of exon model per million reads mapped (FPKMs) data for the relative expression of ARS1 and ARS2 transcripts in the sorghum root tissue are shown in Fig. S1d. Three replicate samples were collected 8, 24, 44, 65, and 96 days after the emergence. RNA sequencing (RNA-seq) data were downloaded from https://phytozome.jgi.doe.gov by searching the gene loci Sobic.005G164300.1 (ARS1) and Sobic.008G036800 (ARS2) (30).

### Study site and field and greenhouse design.

This study was conducted in Mead, NE, USA (41°13′34′′ N, 96°29′18′′ W) in 2015 and 2016 as well as in the greenhouse. The 2015 data were used to refine the experimental design for 2016 and to help identify specific isolates to be used for laboratory assays described below. The data shown in this paper are mainly from the 2016 field season. Sorghum with the RNAi events and the wild-type Tx430 were planted on 3 June 2016. Plots were randomized, and there were six blocks in both years in the field. No additional fertilizer was applied. Supplemental irrigation was carried out on 8, 9, and 15 June due to the hot and dry weather; approximately 1.4 cm of water was added to the field each day.

Sampling in 2016 was conducted on 3 July (4 weeks, seventh-leaf stage), 3 August (9 weeks, boot stage), and 3 September (13 weeks, grain fill stage), and no differences in development were observed between RNAi lines and the WT. Two plants per plot were bulked as one replicate sample. In 2016, five replicates were collected for the two RNAi-event harboring sorghum lines, and seven replicates were collected for the wild type. Sampling of the root, rhizosphere, and bulk soil was performed according to the methods described in a previously published methods paper (63). For the soil near roots, approximately 2 cm of soil around the root surface was collected by shaking the root system inside a 1-gallon ziplock bag. The rhizosphere was the soil that tightly adhered to the roots even after shaking and was removed by vortexing the roots in phosphate buffer. For soil physicochemical analysis, 100 to 125 g sieved soil near roots was analzyed by Ward Lab as described on their website https://www.wardlab.com/submit-a-sample/soil-health-analysis/ (Table S6). The fourth oldest leaf counting from the first emerged leaf was removed, dried, ground, and analyzed for nitrogen content. In 2016, soil was also sampled between rows using a shovel to the depth of 30 cm in a region of the soil that would be less influenced by the sorghum plants. This soil is referred to as “soil between rows.” The panicle from the main stem was harvested from each plant and weighed after drying to compare wild-type and RNAi lines (*N* = 5 to 7). Whole plants at 4 weeks and the main stem of each plant at 13 weeks were also harvested, dried, and weighed.

In the greenhouse, the sorghum harboring each of the two RNAi events and the wild type were planted in pots, replicated four times. The soil used was collected from a field (41°16′13′′ N, 96°67′52′′ W) with extremely low nitrogen concentrations and mixed with sand in a 2:1 ratio in plastic pots measuring 15.2 cm (diameter) by 14.6 cm (height). One week after planting, pots were thinned to contain two plants of similar size. Each pot contained one genotype. Three different concentrations of nitrogen (N100, 11.5 mM added N; N50, 5.75 mM added N; and N10, 1.15 mM added N) were added as a modified Hoagland solution (64) to corresponding pots after 2 weeks from the day of planting. Sampling was carried out 2 months from the planting, which roughly corresponded to the time when differences in β-diversity were observed in field experiments (9 weeks). Pots contained sorghum plants with both RNAi events and wild-type Tx430. The endosphere and rhizosphere samples were collected as described earlier (63), and bulk soil was collected for chemical analysis.

### DNA extraction and amplicon-based 16S rRNA gene analyses.

DNA was extracted using MO BIO PowerPlant Pro-htp and the MO BIO PowerSoil-htp kits (MO BIO, Carlsbad, CA) and processed as described in reference 63. To characterize the microbial populations in samples, the 16S rRNA gene primers 515f and 806r were used to amplify the V4 region of the 16S rRNA gene at the University of Minnesota Genomics facility using their published protocol (65). Sequencing was performed using paired-end 300-base reads on an Illumina MiSeq. For root samples, sequencing was performed with the inclusion of peptide nucleic acid (PNA) blockers (66) to reduce the amount of plant plastid and mitochondrial sequences.

Bioinformatic processing of the sequence data was performed using a combination of QIIME (67, 68) and USEARCH (version 10.0.240) with the UPARSE pipeline (69). Briefly, after demultiplexing, the paired-end reads were merged with error correction using USEARCH. The maximum number of mismatches in the alignment was set at 10 base pairs, and minimum percentage of identity in the alignment was set to 80% due to the long overlaps obtained with 2 by 300-bp V4 region using the MiSeq platform. Primers were removed from the merged sequences. Next, quality filtering was conducted to remove the low-quality reads by setting the maximum number of expected errors (E_max) to 1 as the threshold. The quality-filtered sequences were then deduplicated to find the unique sequences and create the input sequences of the 97% OTU clustering. Then, 97% identity was used as the threshold for OTU clustering. In this step, the minimum abundance (minisize = 2) was set to discard singleton unique sequences and to remove chimeras. Finally, an OTU table was created. Taxonomy was assigned using the Ribosomal Database Project classifier (RDP) (70) and the Greengenes 13_8 database (71). RDP classifier was trained by using marker gene reference databases of Greengenes (13_8 [most recent], 97_otu_taxonomy.txt, and 97_otus.fasta). Plastid and mitochondrial sequences were removed from the analysis. For the α-diversity analyses, the resulting OTU table was rarefied to a fixed number of reads per sample in the data set. Rhizosphere, soil, and root samples were rarefied to 7,681, 11,153, and 334 sequences for the 2016 field data. Rhizosphere and root samples were rarefied to 1,349 and 600 sequences for the greenhouse data. For the β-diversity analyses, the OTU table was separated by the sample type and year for downstream analysis. For statistical analysis between groups, the Bray-Curtis distance matrix was calculated using rarified data for PERMANOVA with the adonis() and anova() function in vegan package (72) v2.4.5 in R studio. The canonical analysis of principal coordinates (CAP) was performed using the function capscale() in the vegan package, and plots were visualized by ggplot2 package (73) v2.2.1 in R studio using R (v3.5.1). Bray-Curtis dissimilarity indexes were used to perform the CAPs. The R and python codes can be found at https://github.com/SchachtmanLab/Transgenic-sorghum-sorgoleone.

### Network analysis.

Co-occurrence networks were constructed using only positive correlations between OTUs for each sampling time for wild-type sorghum and the RNAi lines. Only OTUs that occurred in at least 12 of the 17 samples were included in the analysis, which was defined as the core OTU with a 70% threshold. Equal numbers of replicates (*n* = 7) of the wild type and RNAi lines were used for analysis by randomly selecting the RNAi lines (74). Spearman correlation was performed for the absolute read counts of the different samples of WT or with RNAi events by using SparCC (75). At the same time, pseudo-*P* values were calculated via a bootstrap procedure with 100 shuffles to determine the significance of the correlation. To simplify the network and reduce the false-positive rate, a Spearman’s cutoff ρ of >0.8 and a *P* value of ≤0.01 was chosen to highlight the strongest correlations among OTUs in the soil near roots or rhizosphere samples of wild-type or RNAi event sorghum at 4 weeks, 9 weeks, and 13 weeks after planting. The network visualization and calculations of node degree, betweenness centrality, closeness centrality, and clustering coefficient were performed using the R package igraph (76) and ggraph (77) with the Fruchterman-Reingold layout algorithm (76).

### Potential nitrification rates of soil and rhizosphere.

The method for determining potential nitrification rate (PNR) was adapted from Hart et al. (78). Briefly, 18.75 g of soil near roots was placed in a 250-ml flask and mixed in 125 ml of a liquid containing 1.5 mM NH_4_^+^ and 1 mM PO_4_^3−^. The flasks were incubated at 22°C in a shaker rotating at 200 rpm. Three soil-near-root samples per flask (3 × 5 ml) were taken after 2 h, 4 h, 22 h, 24 h, 46 h, and 70 h of incubation at room temperature by withdrawing 5 ml of the slurry from each flask and transferring to 15-ml conical tubes and assayed. Three replicates were used for each RNAi event (RNAi no. 1 and RNAi no. 2), four replicates for WT, and two replicates of soil between rows at each sampling time. Slurry samples were centrifuged at 4,000 × *g* for 10 min at 4°C, and supernatant was analyzed for NO^3−^ N content using a nitrate/nitrite colorimetric assay kit (Cayman Chemical Co., MI). The potential nitrification rates were determined by linear regression of NO^3−^ N generation over the incubation time as described by Hart et al. (78).

### Response of microbial isolates to sorgoleone.

R2A medium was used to culture bacteria from the rhizosphere, root, and soil samples. Single colonies were identified by sequencing the 16S rRNA amplified by a pair of primers 27F/1492R (27F, 5′-AGAGTTTGATCCTGGCTCAG-3′; 1492R, 5′-GGTTACCTTGTTACGACTT-3′) and sequences were searched in the RDP database to verify the identity of the isolate. The differential abundance analysis for the absolute abundance of the OTUs between sorghum with RNAi events and wild type was determined by using DESeq2 (79). Twenty-nine isolates from the culture experiment were chosen based on genus or family level similarity based on the OTUs in DESeq analysis (Table S2) to test for growth response to sorgoleone. Among these 29 isolates, 18 isolates were cultured from sorghum with RNAi events and the wild type from the experimental field in Mead, NE, in 2015, four were cultured from an alkaline site in the Sandhills of Nebraska, and four were cultured from another field in Mead, NE. These 29 colonies included one nitrifying bacterium Nitrosomonas europaea (ATCC 19718) and two isolates belonging to the *Methylophilaceae* family. Growth curves were performed in R2A liquid medium containing 0.025 mM sorgoleone or no added sorgoleone with methanol (in which the sorgoleone was dissolved) as a control. Three different concentrations of sorgoleone were tested, including 0.00156 mM, 0.003 mM, and 0.025 mM, in preliminary experiments using a 96-well-plate format and a plate reader. Each well contained 160 μl of bacterial culture. The concentration of 0.025 mM sorgoleone was used for the final experiments because it was the lowest concentration that showed repeatable and clearly distinguishable impacts on certain bacteria based on time course of optical density at 600 nm (OD_600_) growth curves. Each growth assay was replicated 3 times for each treatment in 96-well sterile plates with a 160 μl volume in each well. Plates were incubated at 23°C shaking at 220 rpm. Optical density at 600 nm was measured during a 50-h time course using a Synergy 2 multidetection microplate reader (BioTek) at 2-h intervals during the first 24 h and 6-h intervals after 24 h. For two isolates belonging to *Methylophilaceae*, M9 minimal liquid medium was used. A recombinant strain of *N. europaea* was created by introducing an expression plasmid vector for the Vibrio harveyi *luxAB* genes (32). Liquid P-medium (32) was used for the growth of *N. europaea*, and response to sorgoleone was quantified by measuring the bioluminescence readings (80). When calculating the percentage of inhibition due to sorgoleone, the 48-h growth point was used with the formula [(OD_without sorgoleone_/OD_with sorgoleone_) × 100] − 100, except for *Bacillus* no. 5 and *Bacillus* no. 4, which grew at a faster rate and therefore the 4-h time point was used. The OD_600_ data were used to determine whether sorgoleone inhibited or stimulated growth using Welch’s *t* test due to unequal variance between sorgoleone-treated samples and control samples. *P* values from one-sided tests were converted to *q* values to control for false-discovery rates.

### Data availability.

All raw sequence data were deposited to NCBI under the BioProject accession number PRJNA637774.
