# Supplementary figures and images for: The Sorghum bicolor Root Exudate Sorgoleone Shapes Bacterial Communities and Delays Network Formation
Source: mSystems. 2021 Mar 16;6(2):e00749-20. doi: 10.1128/mSystems.00749-20 (PMC8546980; doi:10.1128/mSystems.00749-20)

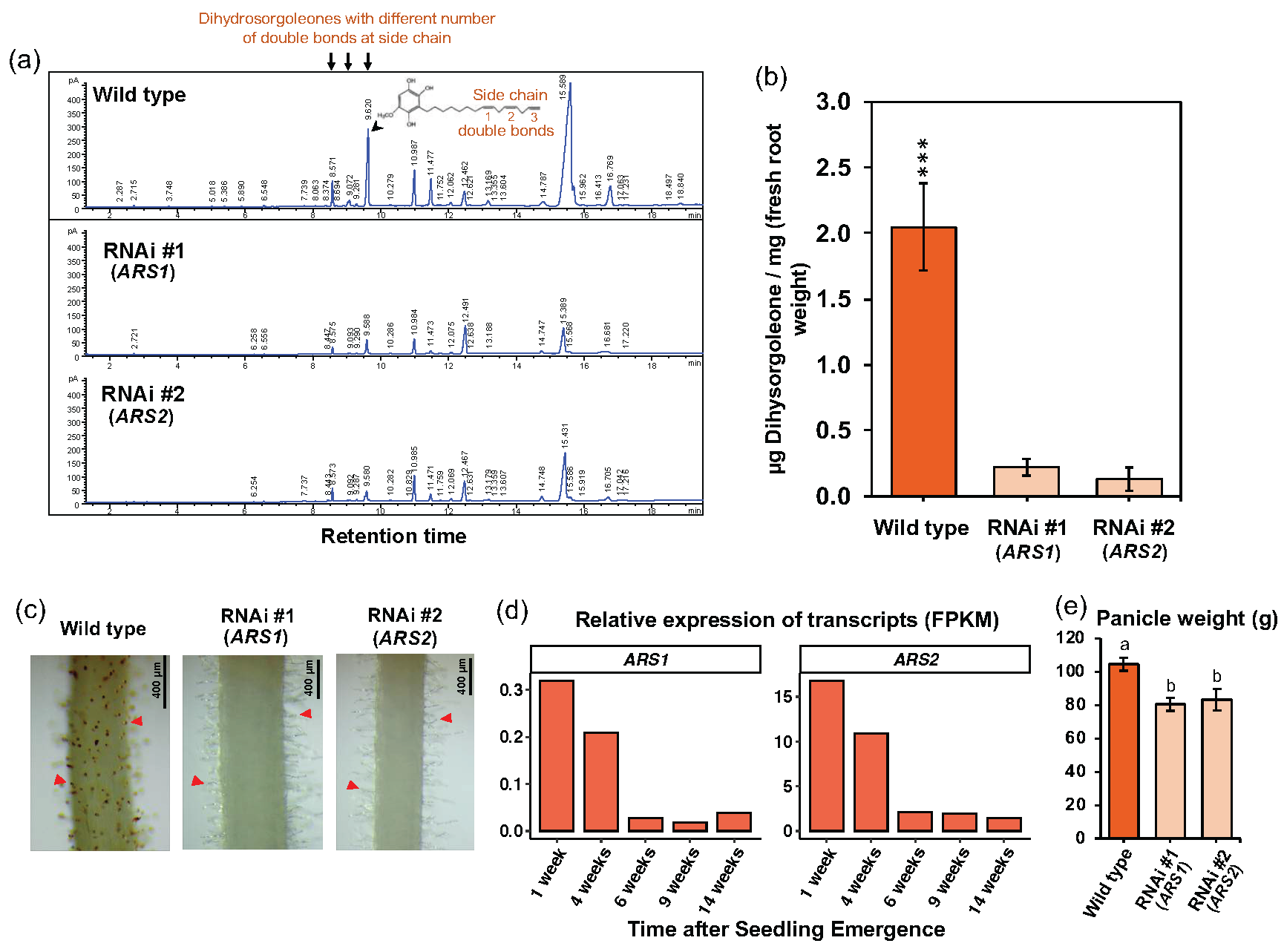

Supplement: FIG S1 [file msystems.00749-20-sf001.tif]

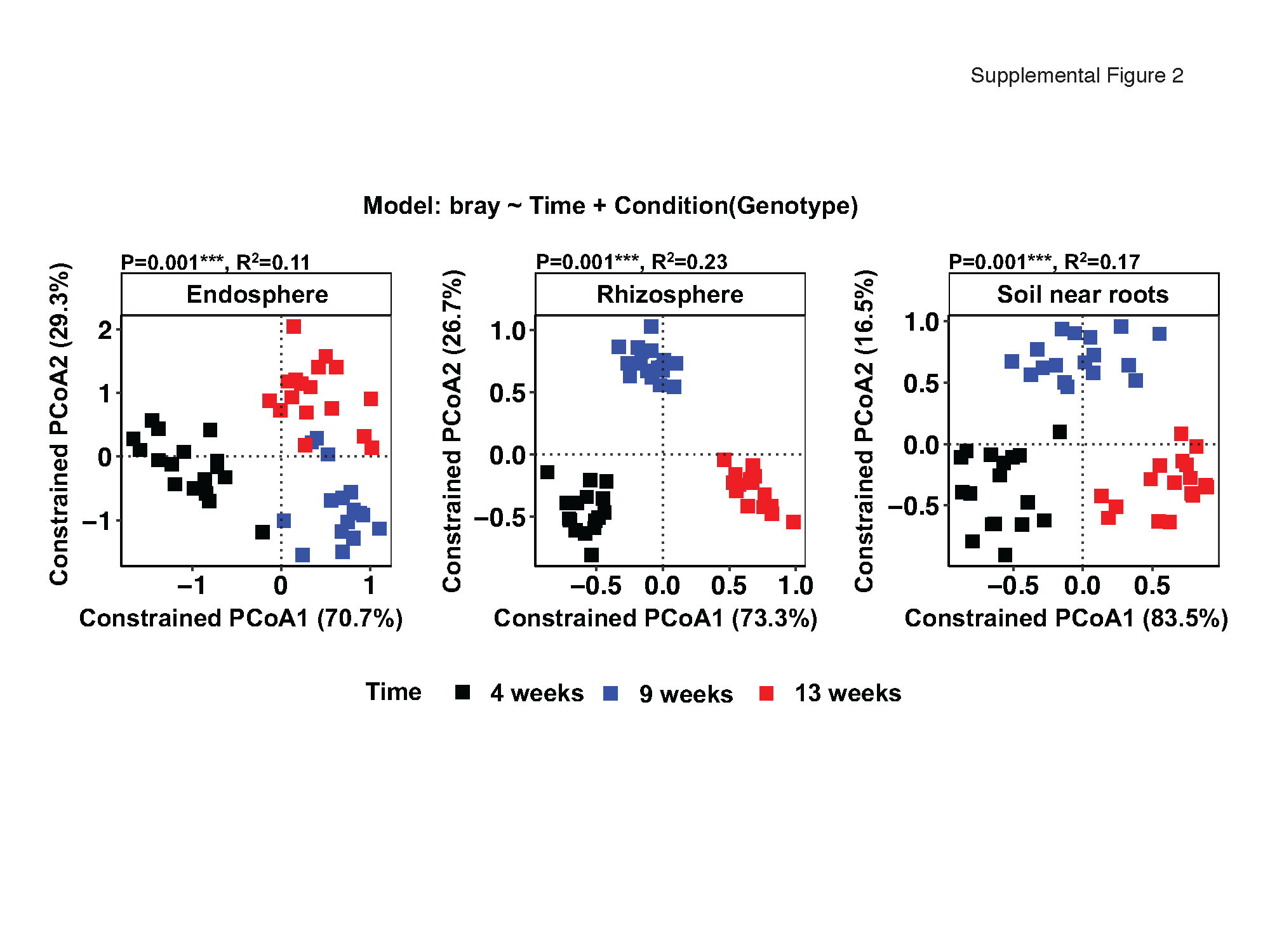

Supplement: FIG S2 [file msystems.00749-20-sf002.tif]

(a)

## Field 2016

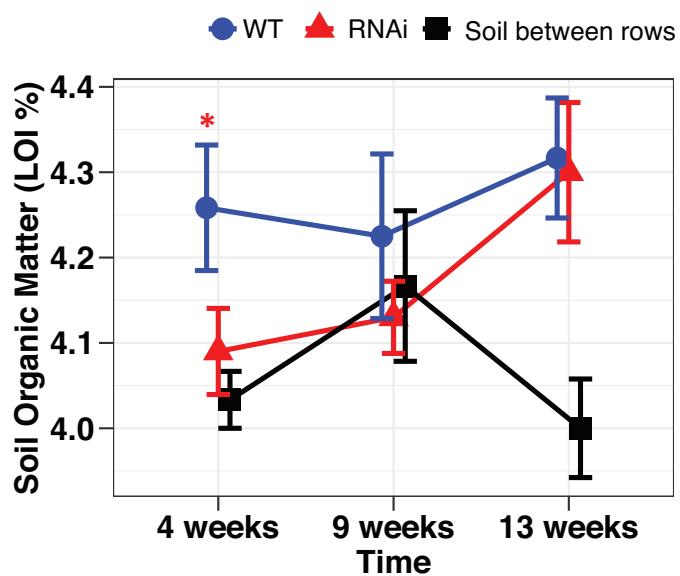

(b)

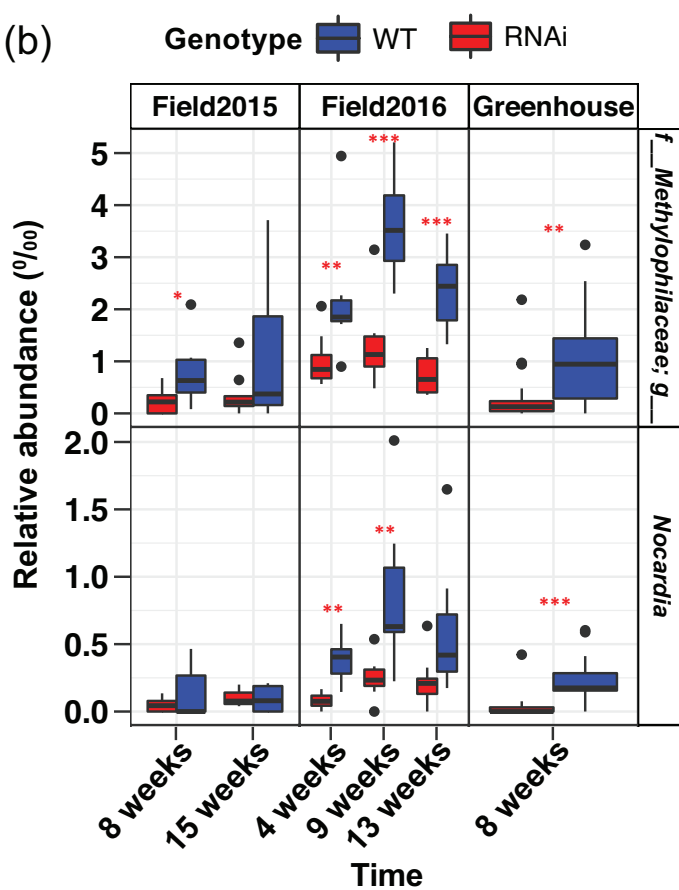

Supplement: FIG S3 [file msystems.00749-20-sf003.pdf]

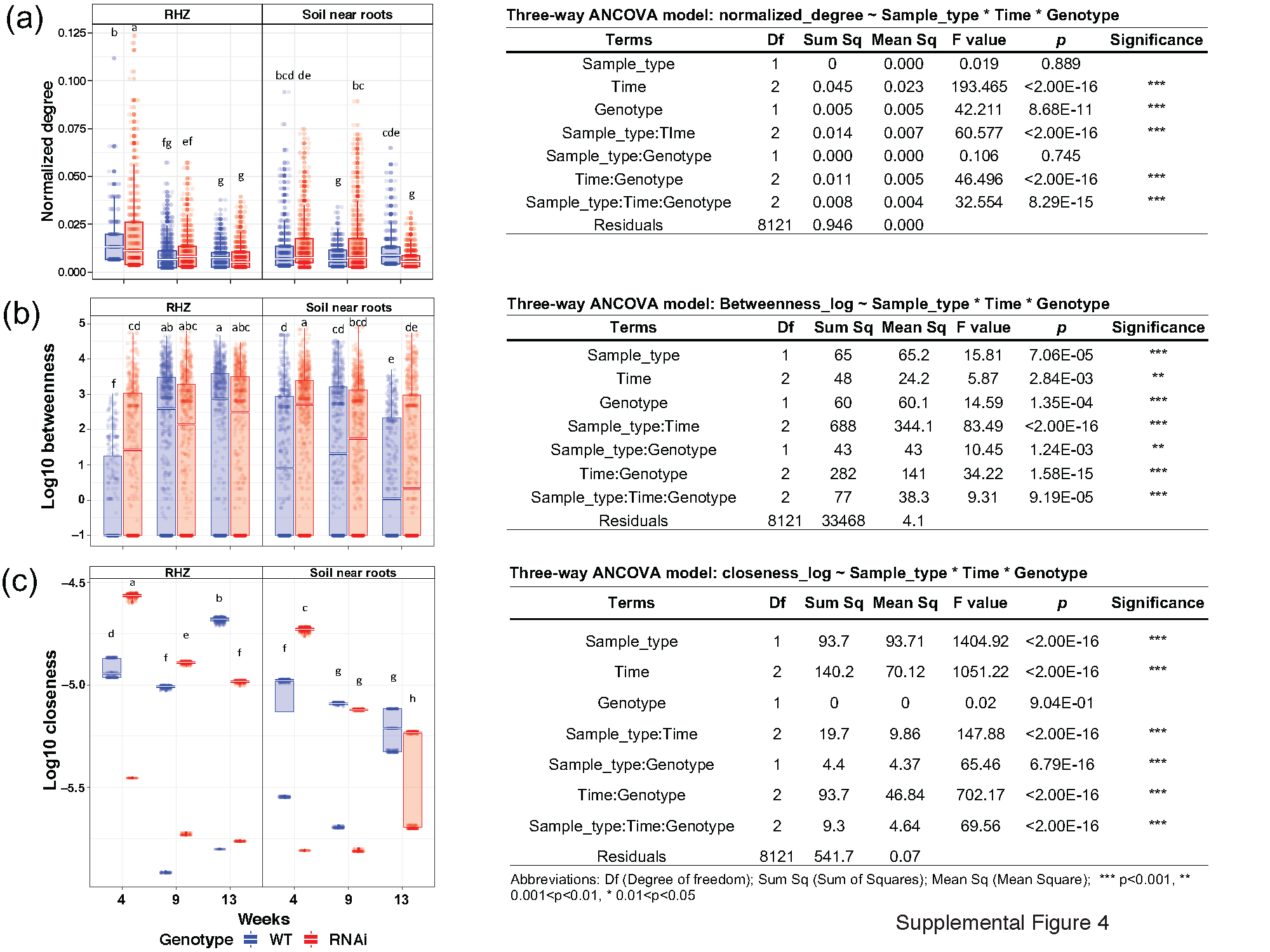

Supplement: FIG S4 [file msystems.00749-20-sf004.tif]
